# Clinical observations of high-flow nasal cannula oxygenation in endobronchial ultrasound-guided transbronchial needle aspiration: a randomized controlled study

**DOI:** 10.3389/fmed.2025.1634020

**Published:** 2025-10-08

**Authors:** Fangli Yue, Xinyuan Shi, Huan Zhang, Shiyu Yu, Min Fu, Yaxin Wei, Hongyi Xiao, Yuqi Zhong, Fanceng Ji, Peihe Nie

**Affiliations:** ^1^Department of Anesthesiology, Weifang People’s Hospital, Weifang, China; ^2^Department of Anesthesiology, The First Affiliated Hospital of Shandong Second Medical University, Weifang, China; ^3^School of Anaesthesiology, Shandong Second Medical University, Weifang, China

**Keywords:** hypoxia, high-flow nasal cannula oxygenation, nasopharyngeal tube oxygenation, endobronchial ultrasound-guided transbronchial needle, intravenous anesthesia

## Abstract

**Purpose:**

The purpose of this study was to compare the clinical effects of high-flow nasal cannula oxygenation and nasopharyngeal tube oxygenation in endobronchial ultrasound-guided transbronchial needle aspiration.

**Patients and methods:**

A total of 81 patients were enrolled in this study. The patients were randomly divided into two groups: the high-flow nasal cannula oxygenation group (HFNC group, *n* = 41) and the nasopharyngeal tube oxygenation group (NPT group, *n* = 40). The HFNC group was given high flow oxygen (oxygen flow rate 45 L/min). In the NPT group, the 6.0 mm ID nasopharyngeal tube was placed and connected to the anesthesia machine’s oxygen port for oxygen inhalation, and an oxygen flow rate of 6 L/min was used. The primary outcome was the incidence of hypoxia. The secondary outcomes measured included the treatment measures used for hypoxia (such as jaw lifting or mask-assisted ventilation). The hemodynamic changes at various time points, along with PaO_2_ and PaCO_2_ values from arterial blood gas analysis, were documented for this and the occurrence of adverse events were recorded.

**Results:**

The incidence of hypoxia in the HFNC group was significantly lower than that in the NPT group (4.9% vs. 25.0%) (*P* = 0.011). The incidence of jaw lifting and mask-assisted ventilation intervention for hypoxia in the HFNC group was significantly lower than that in the NPT group (*P* < 0.05). At T3, the PaO_2_ of the HFNC group was significantly higher than that of the NPT group (*P* < 0.001); PaCO_2_ of the HFNC group was significantly lower than of the NPT group (*P* = 0.015). At T5, the PaO_2_ of the HFNC group was significantly higher than that of the NPT group (*P* < 0.001). There was no significant difference in HR, MAP, SpO_2_, or Ai between the two groups at different time points (*P* > 0.05). There was also no significant difference in the incidence of adverse events between the two groups (*P* > 0.05).

**Conclusion:**

Compared with nasopharyngeal tube oxygenation, the use of high-flow nasal cannula oxygenation in endobronchial ultrasound-guided transbronchial needle aspiration can significantly reduce the incidence of hypoxia and reduce CO_2_ storage, which is worthy of clinical promotion.

**Clinical trial registration:**

https://www.chictr.org.cn, identifier ChiCTR2400085320.

## 1 Introduction

In recent years, the use of endobronchial ultrasound-guided transbronchial needle aspiration (EBUS-TBNA) has led to great progress in the diagnosis and treatment of lung-related diseases (1). At present, most guidelines or expert consensus recommend that sedation anesthesia should be provided for all patients undergoing bronchoscopy without contraindications (2), and increasingly more patients are requiring painless EBUS-TBNA under anesthesia (3). However, unlike other medical procedures with definite sedative effects and high safety, EBUS-TBNA involves stronger airway stimulation and deeper sedation level, making hypoxia more likely (4). EBUS-TBNA oxygenation methods include nasal catheters, nasopharyngeal tubes (NPTs), and oxygen masks. However, NPTs are unsuitable for patients with nasal diseases, and nasal catheters or oxygen masks carry a high risk of hypoxia (29–56%), compromising patient safety (5). High-flow nasal cannula (HFNC) oxygen therapy can input high-flow oxygen, which has the advantages of prolonging safe asphyxia time, reducing the anatomical ineffective cavity of the nasopharynx, and improving the positive end-expiratory pressure (6). At present, HFNC is often used in clinical practice to treat patients with mild to moderate type I respiratory failure (7). Studies have shown that HFNC can significantly improve gas exchange and oxygenation and reduce the risk of hypoxia in patients undergoing bronchoscopy under local anesthesia or mild sedation (8). However, the use of HFNC in deep sedation EBUS-TBNA may be more useful in reducing hypoxia. In this study, HFNC oxygenation was compared with nasopharyngeal tube (NPT) oxygenation to observe the clinical effects of HFNC oxygenation in EBUS-TBNA and to provide a new method for oxygenation during EBUS-TBNA.

## 2 Methodology

The manuscript was written in accordance with the CONSORT statement guideline for a randomized controlled trial.

### 2.1 Study design

This is a randomized controlled study to compare the clinical effects of HFNC oxygenation and NPT oxygenation in EBUS-TBNA under intravenous anesthesia. A total of 81 patients from Weifang People’s Hospital who were scheduled to undergo intravenous anesthesia EBUS-TBNA from July 2024 to September 2024 were selected as the research subjects. The study was approved by the Medical Ethics Committee of Weifang People’s Hospital [KYLL20240204-5] and registered in the Chinese Clinical Trial Registry [ChiCTR2400085320]. This study was carried out in accordance with the principles of the Declaration of Helsinki. All patients provided their written informed consent preoperatively.

### 2.2 Inclusion and exclusion

Inclusion Criteria: Patients undergoing EBUS-TBNA under intravenous anesthesia; ASA II-III; age 18–65 years; and body mass index (BMI) 18.5–28 kg/m^2^. Exclusion Criteria: Patients with a history of asthma; patients with a history of alcoholism; long-term use of opioids or sedatives; patients with a history of severe heart, brain, liver, kidney, or metabolic diseases; patients with a history of nasal polyps, nasal bleeding, nasal trauma, nasal deformity, or nasal inflammation; patients with preoperative low SpO_2_ (defined as SpO_2_ ≤ 92% without oxygen supplementation before operation); or patients with Obstructive sleep apnea hypopnea syndrome (OSAHS).

### 2.3 Randomization and blinding

Random sequences were generated using SPSSAU software, and patients were randomly divided into two groups, namely Group HFNC and Group NPT, in a 1:1 ratio by a researcher. The resulting sequences were placed in opaque, sequentially numbered envelopes. Participating patients were blinded to their group assignments; however, anesthesiologists and outcome recorders were not.

### 2.4 Methods of Anesthesia

All patients were given 2% lidocaine for 15 min of nebulized inhalation and 4 mL of 2% lidocaine for cricothyroid membrane puncture surface anesthesia before surgery. After entering the room, the patient was placed in a supine position and the peripheral veins of the upper limbs were opened. Oxygen inhalation via a mask was set to 6 L/min to improve oxygen reserve, and vital signs such as HR, NIBP, SpO_2_, and Ai (Anesthesia awareness index, ConView YY-105, Zhejiang Yiyang Medical Technology Co., Ltd., China) were monitored by routine monitoring. Dexamethasone 5 mg and glycopyrrolate 0.2 mg were administered intravenously before anesthesia induction. Anesthesia induction involved propofol TCI (plasma target concentration 4 μg/mL) and alfentanil analgesic TCI (plasma target concentration 60 ng/mL). After the patient’s eyelash reflex disappeared, the HFNC group was connected to the high-flow humidified oxygen therapy device (Lifotronic Hi-800, Shenzhen Pumen Company, China). HNFC settings involved a flow rate of 45 L/min, humidification temperature of 37 °C, and FiO_2_ of 100%. In the NPT group, one side of the nostril was selected to insert the NPT with ID 6.0 mm, the flow rate was 6 L/min, the FiO_2_ was 100%, and the nasal lateral end of the NPT was connected to the anesthesia machine for oxygen inhalation. Once the infusion pump showed that propofol and alfentanil had reached their target concentrations, the examiner was instructed to start the operation. Surface anesthesia with 1% lidocaine 5 mL was given by bronchoscopy before reaching the glottis, entering the main bronchus, and bifurcating the carina. Anesthesia was maintained at 40 ~ 60 by adjusting the target plasma concentration of propofol, with increases or decreases of 0.25 μg/mL as appropriate. The target plasma concentration of alfentanil was maintained at 60 ng/mL. Propofol and alfentanil were discontinued at the end of surgery. When SpO_2_ < 90%, the jaw was lifted first and mask-assisted ventilation was not changed for 30 s. If the HR was <50 times/min, the patient was given 0.5 mg of intravenous atropine and 6 mg of ephedrine when the mean arterial pressure (MAP) was <65 mmHg.

### 2.5 Observation indicators

Primary Outcome: Incidence of hypoxia (defined as SpO₂ < 90% for 10 s) (4, 9). Secondary outcomes involved management measures specific to hypoxia (jaw lifting or mask-assisted ventilation). HR, MAP, SpO_2,_and Ai were recorded when entering the operation room (T0), after induction (T1), 5 min after the operation began (T2), 10 min after the operation began (T3), 15 min after the operation began (T4), at the end of the operation (T5), and after recovery (T6) to evaluate safety. Arterial blood gas analysis indexes were taken at T0, T3, and T5. Dosage of propofol and alfentanil, operation time, recovery time (time to open eyes after the end of operation), occurrence of intraoperative adverse events (cough, malignant arrhythmia, or airway spasm), surgeon assessment of examination conditions (the postoperative digital analog scale was used to score examination conditions with a total of 1 ~ 10 points, 1 being very dissatisfied and 10 being very satisfied), and the general condition of the patient were also assessed.

### 2.6 Statistical analysis

According to the results of the pre-test, the incidence of hypoxia in the HFNC and NPT groups was 4.0% (1/25) and 30.0% (6/20), respectively. Based on *α* = 0.05, 1 − *β* = 0.9. The ratio of HFNC and NPT group patients was 1:1, and the sample size was calculated using PASS 15 software. For analysis, 39 were needed per group; considering a dropout rate of 10%, a total of 87 patients were needed for inclusion.

The data were analyzed by SPSSAU. The obtained data were first tested for normal distribution and homogeneity of variance. The measurement data were expressed as mean ± standard deviation if they obeyed the normal distribution, and the independent sample *T* test was used for comparison between groups. Repeated measures analysis of variance was used for intra-group comparison, and pairwise comparison was corrected by Bonferroni. The continuous variables with non-normal distribution were expressed as median (interquartile range) [M (Q1–Q3)], and the rank sum test was used for comparison between groups. The count data were expressed as a percentage, and the chi-square test was used for comparison between groups. Effects were evaluated by Risk ratio (RR) and its 95% confidence interval (CI), and the statistical significance level of all the above tests was defined as a probability value of less than 0.05.

## 3 Results

### 3.1 Patient condition

**Figure 1:**
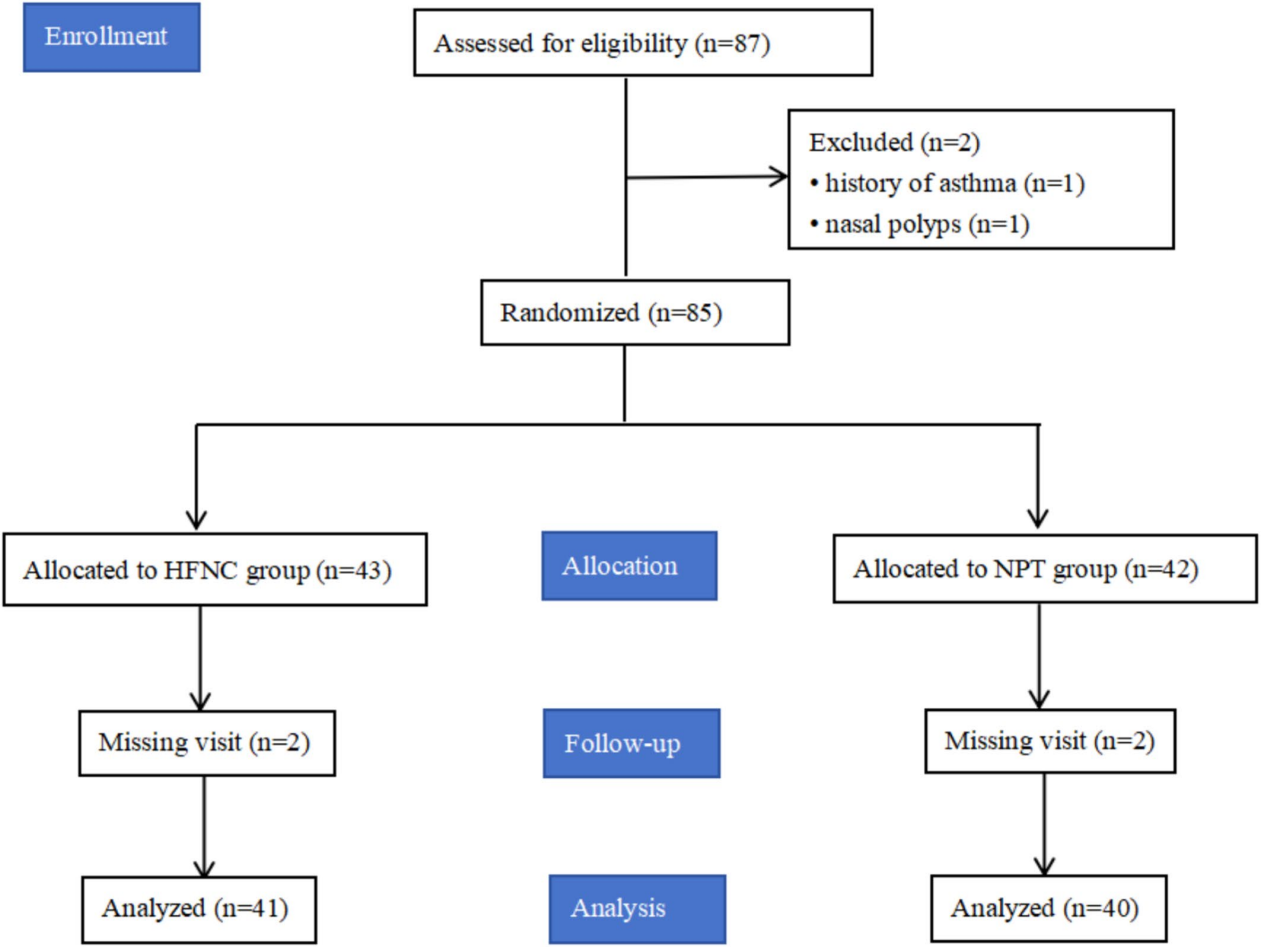
Patients enrollment diagram.

A total of 87 patients were included in this study, and six patients were excluded (one with a history of asthma, one with nasal polyps, and four lost to follow-up). Finally, 81 patients were included in the statistical analysis. Please see Figure 1 for more information.

### 3.2 Baseline and perioperative characteristics

The age, sex ratio, weight, BMI, ASA grade, operation time, and anesthetic drug dosage of the two groups were summarized and analyzed. There was no significant difference in the above information (Table 1).

**Table 1:** General information of patients.

Variables	Group HFNC (*n* = 41)	Group NPT (*n* = 40)	*P* value
Age [years, x̄ ± S]	54.28 ± 9.69	52.57 ± 8.50	0.413
Sex (male/female, *n*)	28/13	21/19	0.146
Weight (kg, x̄ ± S)	66.43 ± 9.90	64.81 ± 11.75	0.480
BMI (kg/m^2^, x̄ ± S)	24.28 ± 2.80	24.32 ± 3.97	0.961
ASA [*n* (%)]			0.675
II	39 (95.1)	37 (92.5)	
III	2 (4.9)	3 (7.5)	
Duration of surgery [min, M (Q1 ~ Q3)]	34 (26, 46)	35.43 (27, 45)	0.564
Propofol dosage [mg, M (Q1 ~ Q3)]	337 (260, 390)	335 (245, 411)	0.720
Alfentanil dosage [mg, M (Q1 ~ Q3)]	1.75 (1.49, 2.13)	1.85 (1.51, 2.10)	0.880

Data are presented as mean (SD), median (interquartile range), or *n* (%).

HFNC, high-flow nasal cannula; NPT, nasopharyngeal tube; BMI, body mass index; ASA, American Society of Anesthesiologists.

### 3.3 Primary outcome

The incidence of hypoxia in the HFNC group was significantly lower than that in the NPT group (4.9% vs. 25.0%) (*P* = 0. 011) (Table 2).

**Table 2:** Primary, and secondary outcomes.

Variables	Group HFNC (*n* = 41)	Group NPT (*n* = 40)	Risk ratio (95% CI)	*P* value
Incidence of hypoxia [*n* (%)]	2(4.9)	10 (25.0)	0.20 (0.05–0.78)	0.011*
Hypoxia interventions [*n* (%)]				
Jaw lifting	2 (4.9)	10 (25.0)	0.20 (0.05–0.78)	0.011*
Mask-assisted ventilation	0 (0)	7 (17.5)	0.00 (0.00–0.59)	0.005*
Satisfaction score of EBUS [x̄ ± S]	8.54 ± 1.12	6.13 ± 1.92	–	0.013*
Recovery time [min, M (Q1 ~ Q3)]	8 (7, 9)	8 (7, 9)	–	0.807
Occurrence of adverse events during operation [*n* (%)]				
Cough	29 (70.7)	27 (67.5)	1.05 (0.79–1.39)	0.813
Malignant arrhythmia	0(0)	0(0)	–	–
Airway spasm	0(0)	1(2.5)	0.00 (0.00–4.93)	0.308

Data are presented as mean (SD), median (interquartile range), or *n* (%). *Significance difference in comparison with control group (*P* < 0. 05).

HFNC, high-flow nasal cannula; NPT, nasopharyngeal tube.

### 3.4 Secondary outcomes

The incidence of jaw lifting intervention for hypoxia in the HFNC group was significantly lower than that in the NPT group (4.9% vs. 25.0%) (*P* = 0.011). The incidence of mask-assisted ventilation intervention in the HFNC group was significantly lower than that in the NPT group (0 vs. 17.5%) (*P* = 0.005). The satisfaction score of microscopic examination conditions in the HFNC group was significantly higher than that in the NPT group (*P* = 0.013). There was no significant comparison of recovery time between the two groups (*P* = 0.807). There was no significant difference in the incidence of adverse events between the two groups (*P* > 0.05) (Table 2).

### 3.5 Arterial blood gas analysis indicators

At T3, the PaO_2_ of the HFNC group was significantly higher than that of the NPT group (*P* < 0. 001); the PaCO_2_ of the HFNC group was significantly lower than that of the NPT group (*P* = 0.015). At T5, the PaO_2_ of the HFNC group was significantly higher than that of the NPT group (*P* < 0. 001); there was no difference in PaCO_2_ between the two groups (*P* = 0.712). Compared with the T0 time, the PaO_2_ of the HFNC group increased significantly at T3 and T5, while PaCO_2_ decreased significantly in the NPT group at T3 and T5 (*P* < 0. 001). There was no difference in PaO_2_ and PaCO_2_ between the HFNC and NPT groups at T3 and T5 (*P* > 0.05) (Table 3).

**Table 3:** Arterial blood gas analysis outcomes.

Variables	Time	T0	T3	T5	*P*_12_ value	*P*_13_ value	*P*_23_ value
PaO_2_ [mmHg]	Group HFNC (*n* = 41)	85.60 ± 20.62	250.30 ± 99.40	263.37 ± 106.42	<0.001*	<0.001*	0.695
	Group NPT (*n* = 40)	82.00 ± 11.09	146.15 ± 71.85	140.68 ± 63.63	<0.001*	<0.001*	0.669
*P* value		0.056	<0.001*	<0.001*			
PaCO_2_ [mmHg]	Group HFNC (*n* = 41)	36.25 ± 4.26	53.61 ± 8.13	56.34 ± 8.05	<0.001*	<0.001*	0.320
	Group NPT (*n* = 40)	37.47 ± 4.29	60.27 ± 13.14	57.35 ± 15.03	<0.001*	<0.001*	0.157
*P* value		0.289	0.015*	0.712			

Data are presented as mean (SD).*Significant difference in comparison with control group (*P* < 0. 05). *P*_12_ and *P*_13_ represent the *P* values compared with T0 and T3 and T5 respectively; *P*_23_ represents the *P* values compared with T3 and T5.

HFNC, high-flow nasal cannula; NPT, nasopharyngeal tube.

### 3.6 Comparison of monitoring indicators

**Figure 2:**
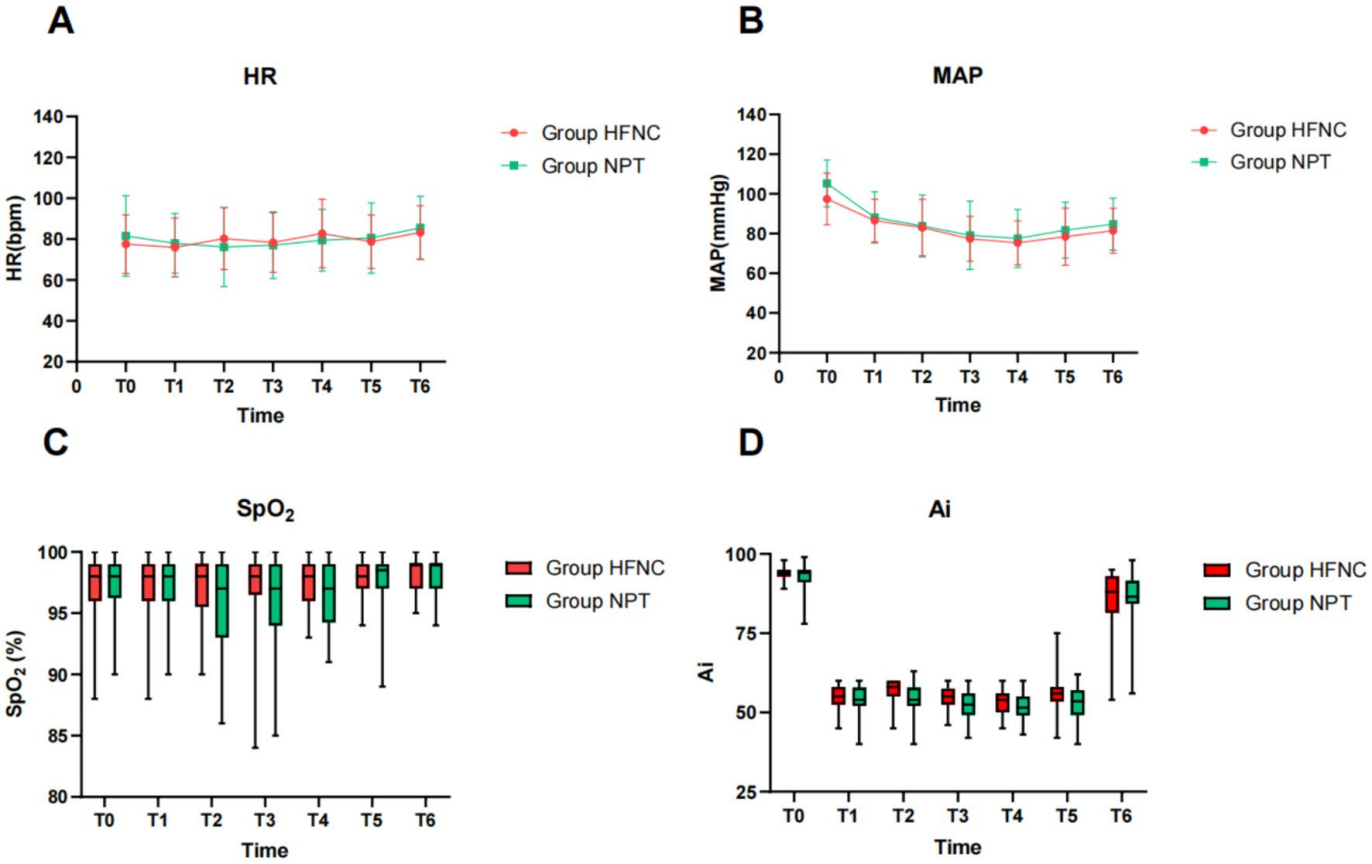
Trend chart of HR, MAP, SpO_2_, and Ai at different time points between the two groups (*P* > 0. 05). HR, heart rate **(A)**, MAP, mean arterial pressure **(B)**, SpO_2,_ oxygen saturation **(C)**, Ai, anesthesia awareness index **(D)**. HFNC, high-flow nasal cannula; NPT, nasopharyngeal tube.

There was no significant difference in HR, MAP, SpO_2_, and Ai at different time points between the two groups (*P* > 0.05) (Figure 2).

## 4 Discussion

The results of this study showed that HFNC oxygenation can significantly reduce the incidence of hypoxia in EBUS-TBNA compared with NPT oxygenation. It may be related to the fact that HFNC can provide accurate and stable inhaled oxygen concentration, transport high-flow oxygen, and have a certain positive end-inspiratory pressure, which is helpful in maintaining a good oxygenation index (10, 11). In addition, anesthetics can cause patients to have respiratory depression or even spontaneously stop breathing. As a new breakthrough in the field of asphyxia oxygenation, HFNC helps to prolong apnea time (12). Luo et al. (13) showed that the use of HFNC during bronchoscopy in patients with hypoxia can effectively reduce the incidence of SPO_2_ < 90% (3.8%). Su et al. (14) found that HFNC oxygen therapy can improve the safety of bronchoscopy. Xu et al. (15) found that 45 L / min flow of HFNC oxygen therapy can significantly reduce the incidence of hypoxemia (3%) in bronchoscopy and reduce the discomfort of patients with oxygen inhalation during operation. In this study, the incidence of hypoxia in HFNC oxygenation in EBUS-TBNA was 4.9%, which was basically consistent with the above research results. Some studies have found that the use of modified HFNC can significantly reduce the incidence of SpO_2_ < 90% during bronchoscopy (12.5%) (16), which is much higher than in our study. This discrepancy may be related to the use of modified single-chamber HFNC and the oxygenation effect being lower than that of a double-chamber. We should also interpret the results carefully, because the diameter of endobronchial ultrasound is larger than that of ordinary bronchoscopy (6.9–7.4 vs. 2.2–6.3 mm). In addition, balloon inflation during lymph node imaging and EBUS-TBNA may lead to increased airway resistance, which increases the risk of hypoxia during surgery (17).

The results of this study showed that the intervention measures specifically for hypoxia in the HFNC group, namely jaw lifting and mask-assisted ventilation, were significantly reduced, which was related to the advantages of HFNC oxygenation in prolonging the time limit of safe asphyxia, reducing the ineffective cavity of nasopharyngeal anatomy and the incidence of hypoxia, improving positive end-expiratory pressure, and increasing lung expansion pressure (18). Some interventions need to interrupt the operation, affecting the operation process and resulting in reduced satisfaction of endoscopists. In addition, the use of NPT reduces patient comfort and satisfaction, and patients with nasal diseases also limit their use (19).

Since EBUS-TBNA is most likely to have complications such as hypoxia, which can be life-threatening in severe cases, it is of great importance to stabilize the patient’s arterial blood gas. The results of this study showed that there was a certain degree of respiratory acidosis and CO_2_ accumulation, but the arterial blood gas analysis indexes PaO_2_ and PaCO_2_ in the HFNC group were significantly better than those in the NPT group. HFNC can clear anatomical dead space through continuous airflow and assist partial alveolar ventilation by the interaction between turbulent supraglottic airflow vortices and cardiogenic oscillations, which contributes to CO_2_ elimination (20). Booth et al. (21) found that HFNC can reduce CO_2_ accumulation in patients with spontaneous breathing without intubation anesthesia, which may be related to the reversal of arteriovenous CO_2_ gradient and the Haldane effect during apnea. Studies have found that the use of HFNC oxygenation during transcatheter aortic valve replacement and transesophageal echocardiography under moderate to deep sedation can reduce the occurrence of hypoxia (22). The above studies suggest that HFNC oxygenation can reduce the risk of CO_2_ accumulation and hypercapnia, which is basically consistent with this study.

The results of this study showed that the changes in hemodynamic indexes, MAP and HR, between the two groups from before anesthesia to the end of surgery were small. These results suggest that the use of HFNC and NPT oxygen in EBUS-TBNA can help stabilize the hemodynamics of patients and reduce the occurrence of adverse reactions. The hemodynamic supply of HFNC to intravenous anesthesia in bronchoscopy has been shown to be more stable (5), which is somewhat different from this study and may be related to its comparison with laryngeal masks.

The results of this study showed that there was no difference in the occurrence of adverse events between the two groups. Cough occurred in both groups, and only one patient had airway spasm, which may be related to the hyperresponsiveness of the airway and the stimulation of the operation. There was no serious cough, however, and airway spasm was also relieved after suspension of the operation and deepening anesthesia.

Limitations of this study: First of all, this study is a single-center study, and the results need to be further verified by multi-center clinical observation. Secondly, this study only included patients with a healthy BMI, and the effect of HFNC on EBUS-TBNA in obese patients needs further study.

## 5 Conclusion

High-flow nasal cannula oxygenation can significantly reduce the incidence of hypoxia and CO_2_ storage compared with nasopharyngeal tube oxygenation in EBUS-TBNA, which is worthy of clinical promotion.

## Data Availability

The original contributions presented in the study are included in the article/supplementary material, further inquiries can be directed to the corresponding author.
